# Fibrous Papule of the Face, Similar to Tuberous Sclerosis Complex-Associated Angiofibroma, Shows Activation of the Mammalian Target of Rapamycin Pathway: Evidence for a Novel Therapeutic Strategy?

**DOI:** 10.1371/journal.pone.0089467

**Published:** 2014-02-18

**Authors:** Jung-Yi Lisa Chan, Kuo-Hsien Wang, Chia-Lang Fang, Wei-Yu Chen

**Affiliations:** 1 Department of Dermatology, Cathay General Hospital, Taipei, Taiwan; 2 Department of Dermatology, Taipei Medical University Hospital, Taipei, Taiwan; 3 Department of Dermatology, School of Medicine, College of Medicine, Taipei Medical University, Taipei, Taiwan; 4 Department of Pathology, School of Medicine, College of Medicine, Taipei Medical University, Taipei, Taiwan; 5 Department of Pathology, Wan Fang Hospital, Taipei Medical University, Taipei, Taiwan; The University of Queensland, Australia

## Abstract

Fibrous papules of the face are hamartomas characterized by stellate-shaped stromal cells, multinucleated giant cells, and proliferative blood vessels in the dermis. The pathogenesis of fibrous papules remains unclear. There is a striking microscopic resemblance between fibrous papules and tuberous sclerosis complex (TSC)-associated angiofibromas. A germline mutation of the *TSC1* or *TSC2* gene, leading to activation of the mammalian target of rapamycin (mTOR) pathway, accounts for the pathogenesis of TSC-associated angiofibromas. Activated mTOR subsequently activates p70 ribosomal protein S6 kinase (p70S6K) and ribosomal protein S6 (S6) by phosphorylation. Rapamycin, a mTOR inhibitor, is effective in treating TSC-associated angiofibromas. The aim of this study was to understand whether the mTOR pathway is activated in fibrous papules. We studied immunoexpressions of phosphorylated (p-) mTOR effectors in fibrous papules, TSC-associated angiofibromas, and normal skin controls. P-mTOR, p-p70S6K and p-S6 were highly expressed in dermal stromal cells and epidermal keratinocytes in fibrous papules and TSC-associated angiofibromas but not in fibroblasts and epidermal keratinocytes of normal skin controls (*p*<0.001). The results suggest topical rapamycin may be a novel treatment option for fibrous papules.

## Introduction

Fibrous papules (FPs) of the face/nose, also known as sporadic angiofibromas (AFs), are very common cutaneous hamartomas which predominantly occur singly on the nose, but occasionally several lesions may exist [1], [2]. Histologically, FPs appear dome-shaped or polypoid with spindle- to stellate-shaped stromal cells, multinucleated giant stromal cells, fibrosis, mononuclear inflammatory cell infiltrate, and proliferative thin-walled vessels with dilated lumina in the dermis [1]–[3]. Collagen fibers commonly show a concentric, laminated arrangement around hair follicles. Hyperkeratosis, hypergranulosis, and flattening of rete ridges are seen in the epidermis. The amount of melanin pigment and the number of melanocytes are commonly increased in the basal layer of the epidermis. In addition to the classic type of FPs, several histologic variants including hypercellular, inflammatory, pigmented, clear-cell, and pleomorphic types, were described [2], [4]. Although dermal stromal cells in FPs were clearly demonstrated to originate from dermal fibroblasts or fibrohistiocytic cells, the underlying molecular mechanisms leading to the formation of FPs remain completely unknown [5]–[7].

FPs are histologically indistinguishable from tuberous sclerosis complex (TSC)-associated facial AFs, also known as adenoma sebaceum, which usually present as multiple lesions on the central face before puberty [1], [8]. The TSC is an autosomal dominantly inherited disorder characterized by the development of hamartomas in multiple organs, particularly the central nervous system, heart, kidneys, and skin [9]. The TSC results from a germline mutation of either the *TSC1* or *TSC2* gene, which respectively encodes the proteins, hamartin and tuberin [10], [11]. Hamartin and tuberin act as a complex to negatively regulate the mammalian target of rapamycin (mTOR) signaling pathway [12]–[16]. Loss of function of the hamartin/tuberin complex leads to activation of mTOR accompanied by phosphorylation of its downstream effectors including p70 ribosomal protein S6 kinase (p70S6K) (at Thr389) and ribosomal protein S6 (S6) (at Ser235/236). Angiomyolipomas (AMLs) are a common renal hamartoma that occur sporadically or in association with the TSC. TSC-associated AMLs were demonstrated by immunohistochemistry to highly express phosphorylated (p-) p70S6K and p-S6 [14], [17], [18]. Similarly, recent studies showed the significance of activation of the mTOR pathway in the pathogenesis of sporadic AMLs, which also expressed p-p70S6K and p-S6 [19], [20]. The pathogenesis of TSC-associated cutaneous hamartomas, including facial AFs and periungual fibromas, was also clarified [21]–[23]. Activation of the mTOR pathway in dermal stromal cells is associated with the formation of TSC-associated cutaneous hamartomas. The understanding has led to therapeutic trials of the mTOR inhibitor rapamycin in the treatment of TSC-associated AFs [24]–[28].

Because FPs and TSC-associated facial AFs bear striking histologic similarities, the mTOR signaling pathway is an ideal and reasonable candidate to explore the pathogenesis of FPs. Understanding of the role of the mTOR pathway in FPs may provide an alternative therapeutic option.

## Materials and Methods

### Tissue samples

Pathology files of Wan Fang Hospital from January 2011 to August 2012 were searched for pathologic diagnoses of FPs and AFs. The pathologic diagnoses of these cases were microscopically reconfirmed by two pathologists (CL Fang and WY Chen). For FP cases, clinical records were reviewed to exclude TSC-associated facial AFs. In total, 40 cases of FPs with enough formalin-fixed paraffin-embedded tissues for immunohistochemistry were included in the study. Ten cases of TSC-associated facial AFs were used as a positive control. Twenty archived facial skin specimens, which were removed for other benign lesions in age-matched patients and contained normal-appearing skin, were also collected. The normal skin part was used for immunohistochemistry. Tissue samples were obtained and used according to protocols approved by the Taipei Medical University-Joint Institutional Review Board (approval no. 201209008). The study was conducted according to the Declaration of Helsinki principles. The institutional review board waived the need for informed consent.

### Antibodies and immunohistochemistry

Antibodies against p-mTOR (Ser2448; 1∶100; Cell Signaling Technology, Danvers, MA, USA), p-p70S6K (Thr389; 1∶100; Assay Biotechnology, Sunnyvale, CA, USA), p-S6 (Ser235/236; 1∶100; Cell Signaling Technology), and p-AKT (Ser473; 1∶100; Cell Signaling Technology) were used. Formalin-fixed paraffin-embedded tissues were sectioned at 5 µm. Immunohistochemical staining was performed on a BenchMark XT autostainer using an iView DAB detection kit (Ventana, Tucson, AZ, USA). Sections were incubated with antibodies for 1 h at 37°C. Appropriate positive and negative controls were included in each assay. Expressions of these antibodies in keratinocytes, dermal stromal cells, and endothelial cells were independently analyzed by all authors. The cases with discrepant scoring results were further analyzed by all authors using multiheaded microscopy. Expression levels were scored semiquantitatively as 0, positive in <1% of cells; 1+, positive in 1%∼25% of cells; 2+, positive in 25%∼50% of cells; and 3+, positive in >50% of cells. To objectively evaluate the expressions of these markers, the authors were blinded to the pathologic diagnoses of the specimens at this stage.

### Statistical analysis

Statistical analyses used Fisher's exact test to determine whether there was a significant difference in the expressions of p-mTOR, p-p70S6K, or p-S6 among FPs, TSC-associated AFs, and normal skin controls. *P*<0.05 was considered statistically significant.

## Results

### Clinical findings

The 40 lesions of FP were excised from 40 patients (10–66 years; median age: 38 years). Fifteen patients were men (37.5%), and 25 were women (62.5%). Lesions ranged in size from 2 to 5 mm in diameter and were present on the nose (38 lesions (95%), neck (1 lesion, 2.5%) and forehead (1 lesion, 2.5%). The majority of the lesions presented as sessile, firm, skin-colored papules. All patients were asymptomatic. The initial clinical diagnosis in 21 patients was FP (52.5%) and the remaining lesions were submitted as skin appendage tumor, soft fibroma, intradermal melanocytic nevus, neurofibroma, verruca, or pyogenic granuloma. No tumor recurrence was noticed in all patients. These were categorized as classic (25 lesions), hypercellular (4 lesions), pigmented (6 lesions), inflammatory (3 lesions), pleomorphic (1 lesion), and clear-cell variants (1 lesion) based on previously proposed criteria [2].

### Immunohistochemical findings

**Figure 1:**
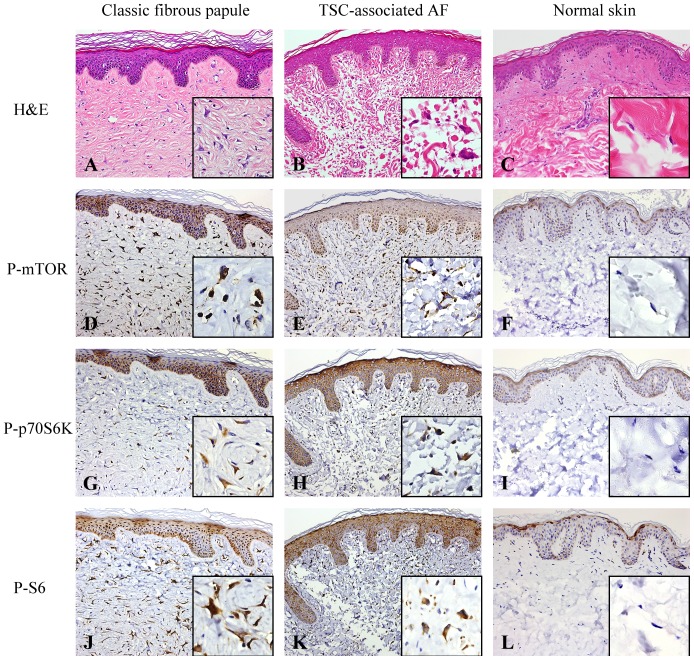
Fibrous papule (FP), tuberous sclerosis complex (TSC)-associated angiofibroma (AF), normal skin (NS), and their expressions of phosphorylated mammalian target of rapamycin (mTOR) effectors. A to C, Stellate-shaped stromal cells and fibrosis in the dermis of FP and TSC-associated AF but not in the dermis of NS. Insets in A and B show the histologic features of stellate-shaped stromal cells in FP and TSC-associated AF compared with fibroblasts in normal skin (inset in C). D to L, Phosphorylated mTOR (p-mTOR), phosphorylated p70 ribosomal protein S6 kinase (p-p70S6K) and phosphorylated ribosomal protein S6 (p-S6) in the dermal stromal cells of FP and TSC-associated AF but not in the fibroblasts of NS. Insets in D to L highlight positive p-mTOR, p-p70S6K and p-S6 immunoreactivity in stellate-shaped stromal cells of FP and TSC-associated AF but not in fibroblasts of normal skin. P-mTOR, p-p70S6K, and p-S6 are also highly expressed in epidermal keratinocytes in FP and TSC-associated AF. Expressions of these markers are limited to granular layers of the epidermis in NS. (A to C, hematoxylin-eosin stain (H&E); original magnification: X200. D to F, P-mTOR immunostaining; original magnification: X200. G to I, P-p70S6K immunostaining; original magnification: X200. J to L, P-S6 immunostaining; original magnification: X200. Insets in D to L, original magnification: x400.)

**Figure 2:**
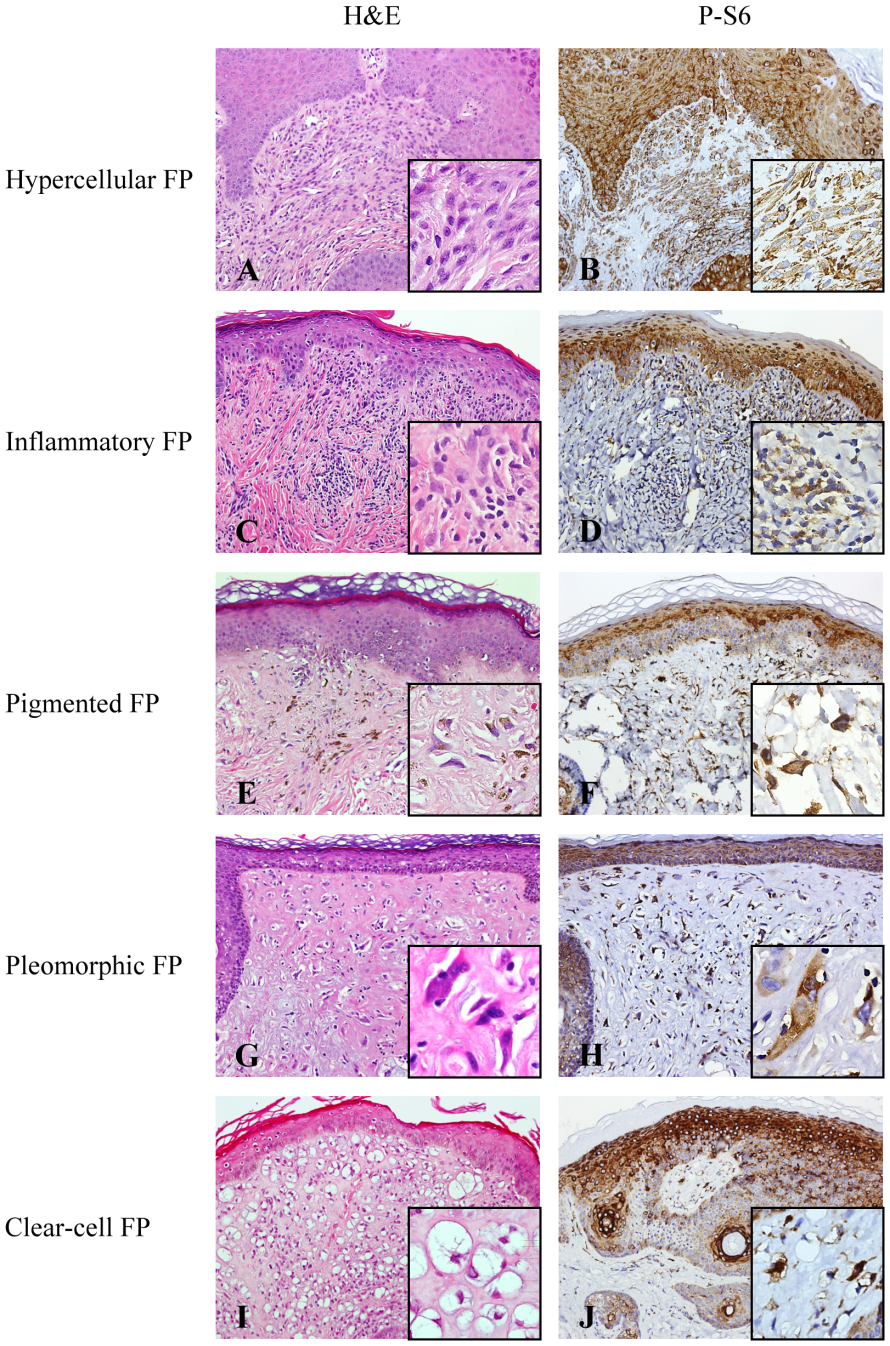
Fibrous papule (FP) variants and their expression of phosphorylated ribosomal protein S6 (p-S6). A and B, Hypercellular FP with closely compacted dermal stromal cells which are immunoreactive for pS6. The epidermal keratinocytes are also positive for p-S6. C and D, Inflammatory FP with stellate stromal cells admixed with lymphocytes in the dermis. The stromal cells and epidermal keratinocytes are immunoreactive for p-S6. E and F, Pigmented FP with melanocyte hyperplasia in the dermoepidermal junction and melanin incontinence. Note p-S6 expression in the stromal cells and epidermal keratinocytes. G and H, Pleomorphic FP with mononucleate and multinucleate stromal cells with enlarged, pleomorphic nuclei. The inset reveals pleomorphic stromal cells with hyperchromatic nuclei and occasional prominent nucleoli. The pleomorphic stromal cells and epidermal keratinocytes are immunoreactive for p-S6. I and J, Clear-cell FP with stromal cells which are immunoreactive for p-S6. The inset in I shows stromal cells with abundant clear cytoplasm. The insets in B, D, F, H, and J highlight positive p-S6 staining in the dermal stromal cells of FP variants. The epidermal keratinocytes are also positive for p-S6. (A, C, E, G, and I: hematoxylin-eosin stain (H&E); original magnification: x200. B, D, F, H, and J: p-S6 immunostaining; original magnification: x200. Insets in A to J, original magnification: x400.)

In all histologic variants of FPs and TSC-associated AFs, p-mTOR, p-p70S6K, and p-S6 exhibited similar staining patterns. Most spindle- and stellate-shaped stromal cells in the dermis of these lesions were immunoreactive for p-mTOR, p-p70S6K, and p-S6 (Figure 1 and 2) (Table I). Among FP specimens, 75% contained nearby normal-appearing skin in which fibroblasts were almost completely negative for these markers (data not shown). Activated mTOR effectors were also observed in nearly all layers of the overlying epidermis of FPs and TSC-associated AFs, with a stronger intensity in granular layers. Endothelial cells rarely expressed p-mTOR, p-p70S6K, or p-S6.

**Table 1:** Expressions of phosphorylated mammalian target of rapamycin effectors in fibrous papule, tuberous sclerosis complex-associated angiofibroma, and normal skin.

			p-mTOR				p-p70S6K				p-S6			
Cell type	Group	N	0	1+	2+	3+	0	1+	2+	3+	0	1+	2+	3+
Dermal stromal cells and fibroblasts	FP*	40	0	0	7	33	0	0	9	31	0	0	3	37
	TSC-associated AF*	10	0	0	1	9	0	0	2	8	0	0	1	9
	NS	20	18	2	0	0	17	3	0	0	16	4	0	0
Epidermal keratinocytes	FP*	40	0	0	6	34	0	0	0	40	0	0	1	39
	TSC-associated AF*	10	0	0	0	10	0	0	1	9	0	0	0	10
	NS	20	0	20	0	0	0	20	0	0	0	20	0	0
Endothelium	FP	40	34	6	0	0	32	8	0	0	36	4	0	0
	TSC-associated AF	10	7	3	0	0	7	3	0	0	7	3	0	0
	NS	20	19	1	0	0	18	2	0	0	17	3	0	0

Abbreviations: FP: fibrous papule, TSC: tuberous sclerosis complex, AF: angiofibroma, NS: normal skin, p-mTOR: phosphorylated mammalian target of rapamycin, p-p70S6K: phosphorylated p70 ribosomal protein S6 kinase; p-S6: phosphorylated ribosomal protein S6.

Expression levels were scored semiquantitatively as 0, positive in <1% of cells; 1+, positive in 1%∼25% of cells; 2+, positive in 25%∼50% of cells; and 3+, positive in >50% of cells.

* *p*<0.001, FP or TSC-associated AF vs. NS.

In contrast, no or only a few fibroblasts in the 20 normal skin controls showed positivity for p-mTOR, p-p70S6K, or p-S6 (Figure 1) (Table I). Endothelial cells in normal skin controls were rarely positive for activated mTOR effectors. Few keratinocytes in the epidermis displayed immunoreactivity for activated mTOR components. These cells were almost completely restricted to granular layers of the epidermis. Percentages of dermal stromal cells and epidermal keratinocytes that were immunoreactive for p-mTOR, p-p70S6K, and p-S6 were significantly higher in FPs and TSC-associated AFs than in normal skin controls (*p*<0.001, Table I). Expressions of activated mTOR effectors in endothelial cells of FPs and TSC-associated AFs did not statistically differ from those in endothelial cells of normal skin controls. P-AKT expression was not observed in dermal stromal cells, endothelial cells and epidermal keratinocytes of all specimens (data not shown).

## Discussion

The designation “fibrous papule” was first proposed in 1965 to describe a dome-shaped, firm, skin-colored papule which often occurred singly on the nose [1]. They may also occur on the forehead, chin, cheeks, and neck [29]. FPs affect middle-aged patients, and both sexes are equally affected [1], [3], [30]. Most patients are asymptomatic, and some may present with bleeding on minor trauma. Patients usually request removal for cosmetic reasons or a microscopic examination is required to confirm the diagnosis. They can be treated by shave excision, elliptical excision, curettage, electrodessication, cryotherapy, or laser therapy [31].

Previous studies of FPs mainly focused on the cell origin of spindle- to stellate-shaped stromal cells and multinucleated giant stromal cells in the dermis [1], [3], [5]–[7], [30]. There has been little progress in understanding the molecular mechanisms of FPs. In one IHC study, loss of expression of tuberin and/or hamartin was detected in TSC-associated AFs but not in FPs [32]. The results seemed to imply that the pathogenesis of FPs is distinct from that of TSC-associated AFs and is not associated with an aberrant TSC1/TSC2 signaling pathway. However, only 36% of TSC-associated AFs showed loss of expression of hamartin and/or tuberin in this study compared to an 83% mutation rate of the *TSC1* or *TSC2* gene in a large cohort study of tuberous sclerosis patients [33]. Immunostaining of hamartin and tuberin does not appear to be sensitive enough to evaluate the functional or mutation status of the *TSC1* or *TSC2* gene. It seems to be too early to exclude the role of aberrant TSC1/TSC2 signaling in the pathogenesis of FPs.

The current study showed considerable overlap in the histologic features and pathogenetic mechanisms between FPs and TSC-associated AFs. In FPs, most spindle- to stellate-shaped stromal cells and multinucleated giant stromal cells in the dermis were immunoreactive for p-mTOR, p-p70S6K, and p-S6, in contrast to negative or rare positive staining in fibroblasts of normal skin controls. Expression patterns of phosphorylated mTOR effectors in the epidermis of FPs were also distinct from those in the epidermis of normal skin controls. Most FPs showed positive staining in all layers of the epidermis, whereas only granular layers of the epidermis were positive for these markers in normal skin controls. There were no statistical differences in the expressions of these markers in endothelial cells between FPs and normal skin controls. The results suggest that dermal stromal cells and/or epidermal keratinocytes, but not endothelial cells, are the most likely cells from which formation of FPs is initiated. The proliferation of blood vessels in FPs may be stimulated by vascular endothelial growth factor (VEGF) produced by mTOR-activated cells. Recent studies revealed a close association between mTOR activation and VEGF production [34], [35]. The mTOR signaling pathway is regulated by multiple inputs including growth factors [13]. In the presence of growth factors, AKT is activated by phosphorylation. P-AKT subsequently phosphorylates TSC2 and finally leads to activation of the mTOR pathway. P-AKT was not detected in dermal stromal cells or epidermal keratinocytes in FPs. These observations indicate that aberrant function of the hamartin/tuberin complex, but not overstimulation of growth factors, may play a major role in the tumorigenesis of FPs. Expressions of p-mTOR, p-p70S6K, and p-S6 were also noted in dermal stromal cells and epidermal keratinocytes in TSC-associated AFs. These observations are consistent with those in a previous study, in which positive p-S6 immunostaining was detected in dermal stromal cells and epidermal keratinocytes of TSC-associated AFs and periungual fibromas [22]. Our study, including two upstream activated effectors of S6, p-mTOR and p-p70S6K, further supports the significance of activation of the mTOR signaling pathway in the pathogenesis of TSC-associated AFs. A previous study of TSC-associated AFs also demonstrated that dermal stromal cells, not epidermal keratinocytes, were most likely the target of a two-hit mutation of the *TSC2* gene based on allelic deletion of TSC2 in dermal stromal cells [22]. Epiregulin, a member of the epidermal growth factor family, was released by dermal stromal cells in TSC-associated AFs [22]. Epiregulin acted as a paracrine factor to enhance keratinocyte proliferation and activate the mTOR pathway in the epidermis. A recent study of the mechanism of somatic second-hit mutations in *TSC1* and *TSC2* genes showed small indels or point mutations in *TSC1* or *TSC2* gene in cultured fibroblasts derived from TSC-associated AFs [36]. The most common second-hit mutation in facial TSC-associated AFs was CC>TT, a well known product of sunlight-induced DNA damage. Because FPs and facial TSC-associated AFs share similar anatomic distributions, histologic features and immunohistochemical findings in activated mTOR effectors, dermal stromal cells in FPs may be the cells which are responsible for the tumorigenesis of FPs. A further molecular analysis such as loss of heterozygosity, fluorescence in situ hybridization and/or DNA sequencing is required to understand whether FPs have any alteration of the *TSC1* or *TSC2* gene in dermal stromal cells, epidermal keratinocytes, or both.

FPs with classic pathologic changes are readily diagnosable based on typical clinical and morphologic features. Several histologic variants including hypercellular, pigmented, inflammatory, clear-cell, pleomorphic, epithelioid, and granular-cell variants were described [2], [4], [37], [38]. No epithelioid or granular cell variant was found in our cases. Although there are wide histologic variations in FPs, activated mTOR was consistently detected. Our findings indicate that these variants truly represent a wide histologic spectrum of a disease with a common pathogenesis.

Through understanding the critical role of activation of the mTOR pathway in the tumorigenesis of TSC-associated hamartomas, therapeutic modalities of the hamartomas are changing. Clinical trials of mTOR inhibitors for treating AMLs, lymphangioleiomyomatosis, and subependymal giant cell astrocytomas are actively being undertaken [39]–[44]. Preliminary data showed that mTOR inhibitors effectively control the growth of hamartomas. Traditionally, TSC-associated AFs are treated by dermabrasion, cryosurgery, curettage, chemical peeling, excision, and laser therapy with a high relapse rate and a risk of scar formation [45]. The efficacy of rapamycin in TSC-associated AFs was recently described in case reports and some small-scale studies [24]–[28], [45]. Because activation of mTOR is evident in FPs, rapamycin may be effective for FPs. Although simple surgical excision is readily performed for a single fibrous papule with an excellent outcome, topical rapamycin may be used in patients with a recurrent or multiple lesions.

In conclusion, our study demonstrated that the pathogenesis of FPs is closely associated with activation of the mTOR signaling pathway. Topical application of an mTOR inhibitor may be a novel therapeutic option for FPs
